# *STAT3* polymorphisms and *IL*-*6* polymorphism are associated with the risk of basal cell carcinoma in patients from northern Poland

**DOI:** 10.1007/s00403-019-01952-7

**Published:** 2019-07-24

**Authors:** Martyna Sławińska, Monika Zabłotna, Jolanta Gleń, Joanna Lakomy, Roman J. Nowicki, Michał Sobjanek

**Affiliations:** 1 Department of Dermatology, Venereology and Allergology, Medical University of Gdańsk, Smoluchowskiego 17 Street, 80-214 Gdańsk, Poland; 2 Department of Patomorphology, Medical University of Gdańsk, Smoluchowskiego 17 Street, 80-214 Gdańsk, Poland

**Keywords:** Basal cell carcinoma, BCC, Gene polymorphism, Interleukin-6, Signal transducer and activator of transcription 3, STAT3

## Abstract

Basal cell carcinoma (BCC) environment consists of stromal and inflammatory cells which produce variety of cytokines, chemokines and growth factors that may affect tumor behavior. One of the cytokines suggested to be involved in the pathogenesis of BCC is IL-6, which is the upstream element of IL-6/JAK/STAT3 pathway. The correlation between polymorphisms of the genes related to this pathway and cancer risk/prognosis have been previously investigated in several neoplasia, but available data concerning BCC are scarce. In the present study, rs1800795 (-174 G/C) *IL*-*6* gene polymorphism and two polymorphisms in the *STAT3* gene, namely rs2293152 (intron 11, C/G) and rs4796793 (-1697, C/G) were assessed in relation to the BCC risk and clinical course. Additionally, IL-6 serum level was assessed in relation to *IL-6* genotype and clinical variables. The study included 254 unrelated patients with BCC and of mean age 70.39 ± 11.43 (69.83 ± 12.32 women, 71.03 ± 10.31 men) and 198 healthy, unrelated age- and sex-matched volunteers. *IL*-*6* and *STAT3* polymorphisms were analyzed using polymerase chain reaction with sequence-specific primers (SSP-PCR). Serum concentration of IL-6 was measured using the ELISA test. We have found that the presence of C allele in rs1800795 *IL*-*6* gene polymorphism was associated with increased risk of BCC (aOR 1.86; 95% CI 1.22–2.84; *p* = 0.004). The presence of CC genotype in *STAT3* rs2293152 polymorphism was associated with increased BCC risk in recessive model analysis (aOR 3.94; 95% CI 1.59–9.77; *p* = 0.003). In contrast, the presence of GC genotype in overdominant model was associated with decreased risk of BCC (aOR = 0.24; 95% CI 0.12–0.49; *p* < 0.0001). The presence of C allele in *STAT3* rs2293152 polymorphism was associated with increased risk of BCC (aOR 1.31; 95% CI 1.01–1.69; *p* = 0.04). The presence of GG genotype in *STAT3* rs4796793 polymorphism was associated with increased BCC risk in recessive model analysis (aOR 3.66; 95% CI 1.33–10.10; *p* = 0.012). The presence of G allele in *STAT3* rs4796793 polymorphism was associated with increased risk of BCC (aOR 1.59; 95% CI 1.01–2.49; *p* = 0.04). IL-6 serum level positively correlated with the tumor size.

## Introduction

The pathogenesis of basal cell carcinoma (BCC) is known to be associated with both genetic and environmental factors [22, 33]. Aberrant activation of Sonic Hedgehog (SHH) pathway seems to play a key role in the genetic background of the tumor development [19, 23]. Nevertheless, it does not explain wide spectrum of clinical presentation, aggressiveness and therapeutic response. Therefore, it has been postulated that additional genetic pathways and immunological factors may influence the incidence and course of the disease [2, 35–37].

The previous studies have shown that the tumor environment consists of stromal and inflammatory cells which produce a variety of cytokines, chemokines and growth factors that may affect the tumor behavior [8, 20, 22, 43].

One of the cytokines suggested to be involved in the pathogenesis of BCC is IL-6 [20]. This pleiotropic cytokine is the upstream element of IL-6/JAK/STAT3 pathway, which is overactivated in many human malignancies [4]. The correlation between polymorphisms of the genes related to this pathway and cancer risk/prognosis have been previously investigated in several neoplasia, but available data concerning BCC are scarce [10, 42, 51].

In the present study rs1800795 (-174 G/C) *IL*-*6* gene polymorphism and two polymorphisms in the *STAT3* gene, namely rs2293152 (intron 11, C/G) and rs4796793 (-1697, C/G) were assessed in relation to the BCC risk and clinical course. Additionally, IL-6 serum level was assessed in relation to *IL*-*6* genotype and clinical variables.

## Materials and methods

### Patients and controls

The study included 254 unrelated patients with BCC and of mean age 70.39 ± 11.43 (69.83 ± 12.32 women, 71.03 ± 10.31 men) and 198 healthy, unrelated age- and sex-matched volunteers. Clinical characteristics of the study group is presented in Table 1. Patients were treated due to primary or recurrent BCC in the Department of Dermatology, Venereology and Allergology, Medical University of Gdańsk, Poland. All cases were confirmed histopathologically. Histopathological diagnoses were made based on the assessment of the hematoxylin and eosin stained sections, according to the classification system of the World Health Organization (WHO) [27]. Single nucleotide polymorphisms (SNPs) were chosen based on their previously documented significance in other neoplasia, on their functional relevance, as well as on the minor allele frequency (MAF) ≥ 0.1 in the Caucasian population (https://www.ncbi.nlm.nih.gov/snp/) [13, 14, 16, 17, 21, 26, 41, 44, 45, 49–51].

**Table 1:** Characteristics of the study group—BCC patients

	Males	Females	Overall group
	*n* = 117 (46.02%)	*n* = 137 (53.94%)	*n* = 254
Age (mean ± SD)			
< 60 years	54.35 (± 5.08)	52.55 (± 5.20)	53.22 (± 5.18)
> 60 years	74.47 (± 7.30)	75.32 (± 8.11)	74.91 (± 7.72)
Mean tumor size (cm)	1.37 (± 0.87)	1.34 (± 1.48)	1.35 (± 1.22)
Diagnosis			
BCC	110 (46.22%)	128 (53.78%)	238
BCC recurrence	7 (43.75%)	9 (56.25%)	16
Number of tumors:			
One tumor	69 (40.12%)	103 (59.88%)	172
Multiple tumors	47 (58.75%)	33 (41.25%)	80
Location			
UV-exposed area	95 (43.78%)	122 (56.22%)	217
Non-UV-exposed area	21 (58.33%)	15 (41.67%)	36
Histopathological subtypes in the studied group			
High-risk (infiltrative, morphoeic, micronodular or basosquamous subtype)	31 (40.79%)	45 (59.21%)	76
Low-risk (superficial or nodular subtype)	26 (54.17%)	22 (45.83%)	48
Undetermined	60 (46.15%)	70 (53.85%)	130

*n* number of patients

Clinical data were collected based on direct interview with the patients and medical records. None of the subjects were organ transplant recipients, none were being treated with immunosuppressive drugs, none suffered from any systemic inflammatory disease or malignancy, none had any signs of infection. The blood samples were collected after obtaining patients’ informed consent, before the surgical procedure. The status of “multiple BCC” was assigned based on previous medical records, medical history or more than one tumor detected during physical examination on hospital admission. High-risk subtypes and low-risk were classified according to WHO classification [27]. If at least one high-risk tumor was detected during the hospitalization of blood collection, the patient was assigned to high-risk category. If more than one tumor was diagnosed during the hospitalization of blood collection, the size of the biggest tumor was considered. Tumors which consisted of two components (high-risk and low-risk) were assigned to high-risk category. Patients with multiple tumors were evaluated towards the clinical and radiological signs of Gorlin-Goltz syndrome and, if necessary, referred to further genetic assessment. The patients with confirmed diagnosis of Gorlin-Goltz syndrome were excluded from the study. Skin surface in anatomical regions considered as chronically exposed to sunlight, namely face, top of the ears, back of the neck, dorsum of the hands and forearms was classified as area exposed to UV radiation. All subjects were exclusively of Polish descent. The study was approved by the local research ethics committee of the Medical University of Gdańsk.

### *IL-6* and *STAT3* genotyping

DNA from the peripheral blood of BCC patients and volunteers were isolated by Blood Mini A&A Biotechnology (A&A Biotechnology, Gdańsk, Poland). *IL*-*6* polymorphism rs1800795 (-174 G/C) and polymorphisms in the *STAT3* gene: rs2293152 (intron 11, C/G) and rs4796793 (-1697, C/G;) were analyzed using polymerase chain reaction with sequence-specific primers (SSP-PCR).

### IL-6 serum level

Serum concentration of IL-6 was measured using the ELISA test (Human ELISA kit, Diaclone SAS, France and Human ELISA kit, Cloud-Clone Corp, TX, USA), following the manufacturer’s instruction.

Concentrations of the IL-6 positively correlated with the age of examined individuals in BCC patients (*p* = 0.02), but not in the control group.

### Statistical analysis

The (χ2) analysis was used to compare the observed number of genotypes with that expected for a population in a Hardy–Weinberg equilibrium. The (χ2) analysis was also employed to test the significance of the differences in the observed alleles and genotypes between groups. A logistic regression model was used to calculate the odds ratios (aORs; adjusted for age and gender) and the 95% confidence intervals (CIs). The Mann–Whitney *U* test was used to compare the mean values, and the correlation was determined using mean Spearman coefficient values. The strength of associations between *IL*-*6* gene rs1800795 polymorphism, *STAT3* gene rs2293152 and rs4796793 polymorphisms and BCC risk was assessed in allelic model, as well as genotype recessive model, dominant model, and overdominant model (Tables 2, 3). Analyses were performed using the Statistica 12.0 software package (StatSoft, Inc., 2015). *p* < 0.05 was considered statistically significant.

**Table 2:** Genotype and allele frequencies for *IL*-*6* rs1800795 in patients with basal cell carcinoma and control subjects

Genotypes and alleles	Controls	BCC	aOR (95% CI), *p*
rs1800795 (G/C)			
Recessive model			
CC	31 (15.66%)	56 (23.05%)	1.54 (0.82–2.92) *p* = 0.05
GC + GG	167 (84.34%)	187 (76.95%)	
Dominant model			
CC + GC	105 (53.03%)	147 (60.49%)	2.46 (1.32–4.56) *p* = 0.50
GG	93 (46.97%)	96 (39.51%)	
Overdominant model			
GC	74 (37.37%)	91 (37.45%)	1.54 (0.82–2.92) *p* = 0.18
CC + GG	124 (62.63%)	152 (62.55%)	
G	260 (65.66%)	283 (58.23%)	**1.86 (1.22–2.84)** ***p*** **= 0.004**
C	136 (34.34%)	203 (41.77%)	

Significant values are highlighted in bold

**Table 3:** Genotype and allele frequencies for *STAT3* rs2293152 and rs4796793 in patients with basal cell carcinoma and control subjects

Genotypes and alleles	Controls	BCC	aOR (95% Cl), *p*
rs2293152 (G/C)			
Recessive model			
CC	20 (10.10%)	65 (26.75%)	**3.94 (1.59–9.77)** ***p*** **= 0.003**
GC + GG	178 (89.9%)	178 (73.25%)	
Dominant model			
CC + GC	125 (63.13%)	148 (60.91%)	0.55 (0.28–1.09) *p* = 0.09
GG	73 (36.87%)	95 (39.09%)	
Overdominant model			
GC	105 (53.03%)	83 (34.16%)	**0.24 (0.12–0.49)** ***p*** **< 0.0001**
CC +GG	93 (46.97%)	160 (65.84%)	
G	251 (63.38%)	273 (56.17%)	**1.31 (1.01–1.69)** ***p*** **= 0.04**
C	145 (36.62%)	213 (43.83%)	
rs4796793 (C/G)			
Recessive model			
GG	19 (9.60%)	54 (22.22%)	**3.66 (1.33–10.10)** ***p*** **= 0.012**
CG + CC	179 (90.04%)	189 (77.78%)	
Dominant model			
GG + CG	98 (49.49%)	140 (57.61%)	1.43 (0.76–2.67) *p* = 0.27
CC	100 (50.51%)	103 (42.39%)	
Overdominant model			
CG	79 (39.90%)	86 (35.39%)	0.78 (0.41–1.50) *p* = 0.46
CC + GG	119 (60.1%)	157 (64.61%)	
C	279 (70.45%)	292 (60.08%)	**1.59 (1.01–2.49)** ***p*** **= 0.04**
G	117 (29.55%)	194 (39.92%)	

Significant values are highlighted in bold

## Results

### *IL*-*6* polymorphism

The *IL*-*6* genotype and allele frequencies in BCC patients and in controls are shown in Table 2. The distribution of the *IL*-*6* genotypes in BCC and control group was not consistent with the Hardy–Weinberg equilibrium. The presence of C allele in rs1800795 *IL*-*6* gene polymorphism was associated with increased risk of BCC (aOR 1.86; 95% CI 1.22–2.84; *p* = 0.004). There were no statistically significant associations between allele and genotype frequencies and tumor size, location, histopathological subtype (high-risk vs. low-risk) and number of tumors diagnosed. We have found no statistical differences between the group of patients with primary and recurrent BCC.

### *STAT3* polymorphisms

Table 3 shows genotype and allele frequencies for *STAT3* rs2293152 and *STAT3* rs4796793 polymorphisms in BCC patients and controls. The distribution of the *STAT3* rs2293152 and rs4796793 genotypes was consistent with the Hardy–Weinberg equilibrium only in the control group.

The presence of CC genotype in *STAT3* rs2293152 polymorphism was associated with increased BCC risk in recessive model analysis (aOR 3.94; 95% CI 1.59–9.77; *p* = 0.003). In contrast, the presence of GC genotype under overdominant model was associated with decreased risk of BCC (aOR 0.24; 95% CI 0.12–0.49; *p* < 0.0001). The presence of C allele was associated with increased risk of BCC (aOR 1.31; 95% CI 1.01–1.69; *p* = 0.04). There were no statistically significant associations between allele and genotype frequencies and tumor size, location, histopathological subtype (high-risk vs. low-risk) and number of tumors diagnosed. We have found no statistical differences between the group of patients with primary and recurrent BCC.

The presence of GG genotype in *STAT3* rs4796793 polymorphism was associated with increased BCC risk in recessive model analysis (aOR 3.66; 95% CI 1.33–10.10; *p* = 0.012). The presence of G allele in *STAT3* rs4796793 polymorphism was associated with increased risk of BCC (aOR 1.59; 95% CI 1.01–2.49; *p* = 0.04).

There were no statistically significant associations between allele and genotype frequencies and tumor size, location, histopathological subtype (high-risk vs. low-risk) and number of tumors diagnosed. We have found no statistical differences between the group of patients with primary and recurrent BCC.

### IL-6 serum level

We have noted significantly increased IL-6 serum levels in BCC patients in comparison with healthy controls (median 3.44; mean 5.59 ± 10.02 pg/ml; range 0.61–101.00 pg/ml vs. median 1.05; mean 1.39 ± 1.28 pg/ml; range 0.08–6.27 pg/ml; *p* < 0.00001). IL-6 serum level positively correlated with tumor size (*p* = 0.005). There were no statistically significant associations between IL-6 serum level and tumor location, histopathological subtype (high-risk vs. low-risk), number of tumors diagnosed. Similarly, we found no statistical differences in IL-6 serum level between the group of patients with primary and recurrent BCC. Additionally, no statistically significant correlations were found between the analyzed IL-6 polymorphism and the IL-6 serum level (Table 4).

**Table 4:** Median serum concentrations of IL-6 for *IL-6* genotypes

IL-6 rs1800795 (G/C) genotypes	Median IL-6 serum concentration (lower/upper quartile) [pg/ml]	
	BCC patients (*n* = 135)	Controls (*n* = 48)
GG	3.48 (2.61–4.93)	1.11 (0.55–1.76)
GC	3.43 (2.45–4.62)	1.08 (0.87–2.34)
CC	3.42 (1.92–7.45)	0.72 (0.27–1.58)

## Discussion

BCC is known to be an immunogenic neoplasia and cytokines seem to play an important role in the tumor pathogenesis [8]. The previous studies showed that Th2 cytokines generally predominate in tumor microenvironment, while Th1 cytokines are more prevalent in regressing BCC [8, 43].

One of the cytokines involved in the pathogenesis of BCC is IL-6. This pleiotropic cytokine was identified as an important link between inflammation and cancer [4]. IL-6 role has been widely investigated in different types of human neoplasia and the studies showed that it may act in an autocrine or paracrine manner, influence the growth of a tumor either directly or indirectly, through modification of tumor environment [53]. IL-6 is also involved in the activation of different signaling pathways, including IL-6/JAK/STAT3 pathway [4]. IL-6, JAK1, JAK2 and STAT3 which are considered main components of this pathway, have been found to be overexpressed in different cancers, including BCC [5, 8, 12, 29, 40]. Similarly, genetic variants of the genes encoding elements of IL-6/JAK/STAT3 pathway were related to the risk, course, prognosis and response to treatment of different neoplasia, but available data concerning BCC are scarce [10, 42, 51].

In normal epidermis IL-6 is expressed predominantly in proliferating keratinocytes [32, 46]. Ultraviolet radiation, apart from inducing DNA photodamage, may also stimulate skin cells to release cytokines, i.e. IL-1, IL-6, IL-10, IL-12, TNF-α [9, 24, 25, 34]. Jee et al. [20] who investigated the possible role of IL-6 in the pathogenesis of BCC on the tumor cell lines concluded that this cytokine is involved in both suppression of apoptosis and stimulation of angiogenesis. According to the study, IL-6-expressing BCC clones (BCC cells transfected with IL-expressing vector) revealed higher DNA synthesis and were resistant to apoptosis. Detailed mechanism of this association remains to be elucidated, but it may be connected with antiapoptotic protein Mcl-1, whose overexpression was shown in IL-6 transfectants. IL-6-induced stimulation of angiogenesis is most probably the effect of increased production of vascular endothelial growth factor (VEGF) and cyclooxygenase-2 (Cox-2).

Another interesting connection concerns IL-6 and SHH pathway. Lesiak et al. [28], in a recently published study, showed UVB-induced expression of SHH proteins, and suggested that UVB radiation may activate noncanonical SHH pathway. As IL-6 is also released from keratinocytes after UV exposure, we hypothesize that it could be considered as one of the factors activating SHH pathway in human epidermis. Although we have not found any reports devoted to this relationship in the context of BCC, studies based on different neoplasia showed that IL-6 may upregulate Shh and Gli-1 expression [31, 38]. Prolonged activation of SHH pathway in keratinocytes may result in the suppression of differentiation and reprogramming cells to resemble interfollicular epidermal progenitor cells, resulting in BCC formation [1, 47].

Increased expression of IL-6 was confirmed on human BCC specimens compared to healthy skin [8, 12, 40]. In a study conducted by Elamin et al. [8] IL-6 level tended to be higher in more aggressive histopathological variants of BCC; however, this correlation was not statistically significant. In the present study IL-6 serum level positively correlated with tumor size (*p* = 0.005), in contrast no correlation with the histopathological subtype was found. The role of IL-6 in the tumor pathogenesis seems to be more complex. Additionally, its serum concentrations may be affected by multiple factors, so these results should be interpreted with caution.

*IL*-*6* gene is located on chromosome 7p21 with an upstream promoter containing 303 base pairs (bp) [3]. The common G/C polymorphism of the *IL*-*6* promoter on -174 position (rs1800795) has a functional relevance. The presence of allele G results in higher transcriptional activity, subsequently leading to higher serum IL-6 concentration [11]. In the present study GG genotype was associated with higher (but not statistically significant) IL-6 serum level concentration than CC genotype, both in the BCC group and controls.

According to the genetic population studies the frequency of -174G allele is higher in non-Caucasian in comparison to Caucasian populations [6, 30]. Clinical significance of this polymorphism has been previously investigated in variety of human neoplasia [50]. Wilkening et al. [42] concluded that the role of *IL*-*6* -174 genotype in carcinogenesis may have tissue-specific significance. For instance, The CC genotype has been associated with better prognosis in patients with ovarian cancer and vulvar cancer [14, 16]. In contrast, the same genotype was found to be a negative prognostic factor in colorectal, breast and lung cancer [13, 17, 26].

To our knowledge, the role of *IL*-*6* -174 polymorphism in the pathogenesis of BCC was previously the subject of two studies, but none of them correlated particular genotypes with IL-6 serum level [10, 51]. Festa et al. [10] investigated *IL*-*6* -174G/C, -634G/C, and -597G/A polymorphisms in a Swedish and Finnish cohort and found no difference for genotype distributions between BCC patients (*n* = 197) and controls (*n* = 540). Similar results concerning the same polymorphisms were showed in a Swedish study (241 BCC patients, 260 controls) by Zhang et al. [51]. In our study, the presence of C allele was associated with increased risk of BCC.

STAT3 is a key component of IL-6/JAK/STAT3 pathway. Persistent activation of STAT3 was detected in many human malignancies and linked to mechanisms of proliferation, invasion of malignant cells, angiogenesis and inhibition of anti-tumor immunity [48].

The studies concerning expression of STAT3 in non-melanoma skin cancer (NMSC), although scarce, also have shown increased expression of these proteins in NMSC cells compared to normal epidermis [5, 29, 39].

*STAT3* gene is located on chromosome 17q21. It has been hypothesized, that SNPs in *STAT3* gene may influence STAT3 expression, activation upon stimulation and contribute to predisposition to inflammatory and neoplastic diseases [45].

In the present study we examined two *STAT3* polymorphisms: rs4796793 (in the promoter) and rs2293152 (in intron 11). Both SNPs were previously investigated in the context of several neoplasia, but the results were inconsistent [45]. For instance, rs4796793 polymorphism was related to parametrial invasion and poor differentiation of cervical cancer [41] and increased risk of breast cancer [52]. In contrast, Jiang et al. [21] found an association between the presence of rs4796793 minor alleles and decreased risk of lung cancer. rs2293152 polymorphism was associated with hepatocellular carcinoma risk, but no with gastric and lung cancer [21, 44, 49].

To our knowledge, this is the first study concerning the role of *STAT3* gene polymorphisms in the pathogenesis of BCC. We have found that the presence of GG genotype (in recessive model) and G allele in *STAT3* rs4796793 polymorphism was associated with increased risk of BCC. The presence of CC genotype (in recessive model) and C allele in *STAT3* rs2293152 polymorphism increased the risk of BCC.

Previous studies explaining the functional relevance of *STAT3* polymorphisms are few [7, 18]. Ito et al. [18] revealed that rs4796793 polymorphism influences STAT3 mRNA level, and correlated CC genotype with lower STAT3 expression. In this study, C allele was more prevalent in patients with metastatic renal cell carcinoma who positively responded to INF-α therapy and was proposed as a marker predicting therapeutic response. This observation could also be applied in further studies concerning BCC patients treated with imiquimod—topical immune response modifier which acts through inducing proinflammatory cytokines, including INF-α [15].

As for rs2293152 located in the 11 intron of *STAT3* gene, we are unaware of any study examining its functional relevance, but it is hypothesized that it may influence the cancer risk by affecting gene transcriptional regulation or the process of mRNA splicing [45].

The present study has some limitations. First, the studied population was selected from one region, so the results may not be representative of other populations. Secondly, the studied group was relatively small, which could influence the analysis statistical power. The third limitation was the retrospective collection of some data, like the previous history of BCC, as not all patients had completed medical records/good knowledge about previously excised skin lesions. As it was difficult to objectively measure the lifetime exposure to sunlight, we had decided to consider sun-exposed vs. non-sun-exposed skin areas instead.

In the light of the presented findings, we suggest that *STAT3* and *IL*-*6* are involved in BCC pathogenesis and the studied polymorphisms may influence the susceptibility of the disease. Increased serum level of IL-6 in BCC patients compared to controls also supports its suggested pathogenetic role. This should encourage further studies on possible therapeutic applications targeting IL-6/JAK/STAT3 pathway.
